# Diagnostic Utility of Neutrophil CD64 as a Marker for Early-Onset Sepsis in Preterm Neonates

**DOI:** 10.1371/journal.pone.0102647

**Published:** 2014-07-17

**Authors:** Jikun Du, Li Li, Yuhong Dou, Peipei Li, Rui Chen, Helu Liu

**Affiliations:** 1 Department of Clinical Laboratory, Shenzhen Shajing Hospital Affiliated of Guangzhou Medical University, Shenzhen, China; 2 Department of Pharmacology, Guangdong Medical College, Dongguan, China; 3 Shenzhen Baoan Maternal and Child Health Hospital, Shenzhen, China; Children’s Hospital Boston & Harvard Medical School, United States of America

## Abstract

**Introduction:**

Neutrophil CD64 has been proposed as an early marker of sepsis. This study aims to evaluate the diagnostic utility of neutrophil CD64 for identification of early-onset sepsis in preterm neonates.

**Methods:**

The prospective study was conducted in a neonatal intensive care unit between November 2010 and June 2011. Preterm neonates in whom infection was suspected when they were <12 hours of age were enrolled. Complete blood count with differential, blood culture, neutrophil CD11b and CD64 measurement were performed. Receiver operating characteristic curve analysis was performed to evaluate the performance of neutrophil CD64 as biomarker of sepsis.

**Results:**

A total of 158 preterm neonates was enrolled, 88 of whom were suspected infection. The suspected sepsis group was of lesser gestational age (*P*<0.001) and lower birth weight (*P*<0.001), compared with controls. The hematologic profiles of the suspected sepsis group were characterized by higher white blood cell count, neutrophil counts and C-reactive protein. The suspected sepsis neonates had significantly higher neutrophil CD64 expression compared with controls. Neutrophil CD64 had an area value under the curve of 0.869 with an optimal cutoff values of 1010 phycoerythrin molecules bound/cell and it had a high sensitivity (81.82%) and negative predictive value (77.4%). The level of neutrophil CD64 was independent of antibiotic therapy within 24 hours after the onset of sepsis in preterm neonates.

**Conclusions:**

Neutrophil CD64 is a highly sensitive marker for suspected early-onset sepsis in preterm neonates. Our study suggests that neutrophil CD64 may be incorporated as a valuable marker to diagnose infection.

## Introduction

Sepsis in neonates is a global problem and is a significant cause of neonatal mortality [1]. Prematurity predisposes to sepsis, given their immature immune system and the added contribution of a variety of risk factors [2]. Even late-preterm neonates have a fourfold higher risk of sepsis than term neonates. Clinical symptoms of neonatal sepsis are subtle, late and nonspecific, particularly in preterm neonates, in whom the onset of sepsis may be acute and clinical course can quickly deteriorate [3].

Early diagnosis of neonatal sepsis is the mandatory prerequisite for timely treatment. Isolation of bacteria from a central body fluid (usually blood) remains the gold standard for definitive diagnosis [4], [5]. However, confirmation of positive cultures requires days, and the sensitivity of the culture method is frequently low. Due to this delay and uncertainty, broad-spectrum antibiotics are administrated to all suspect neonates [6]. The problem of unnecessary exposure to antibiotics in this vulnerable population remains, and promotes emergence of drug-resistant strains and the potential for poor outcomes [7].

Attempts have been made to seek an ideal early marker of neonatal sepsis [8]. C-reaction protein (CRP), hematologic parameters, and cytokines have been used to identify accurately neonates with sepsis. Despite the fact that a majority of cytokine markers have good sensitivity and good specificity, these cytokines have not been adopted for general diagnosis of neonatal sepsis [9]. The limits of cytokines for early diagnosis of sepsis are the time required for the test to become positive, the amount of blood required, and the cost involved. The readily achievable complete blood count and leukocyte differential assays have relatively poor specificity for diagnosis of sepsis [8]. Recently, CRP has been used to increase diagnostic sensitivity and specificity [10], [11]. However, the reported sensitivity and specificity for this marker ranges widely, which make it less than ideal [12]. Therefore, the need persists for improved diagnostic indicators of neonatal sepsis.

A number of cell surface antigens have been used as diagnostic markers of neonatal sepsis [13]. CD64, the high affinity Fc receptor, is normally expressed by monocytes and only weakly on resting neutrophils [14]. Upregulation of CD64 on neutrophils (nCD64) is thought to be a very early step of host’s immune response to bacterial infection, increasing approximately one hour after invasion [15]. This upregulated expression is stimulated by inflammatory cytokines during infection, and occurs in a graded manner dependent on the intensity of the cytokine stimulus, and nCD64 expression is stable for more than 24 hours. Advances in flow cytometric technology have made it possible to quantitate nCD64 rapidly for neonates with precision and minimal blood volumes [16]. However, the expression of nCD64 has not been investigated extensively in preterm neonates with sepsis.

We hypothesized upregulation of nCD64 occurs in sepsis of preterm neonates. Firstly we evaluate the diagnostic utility of nCD64 as an early marker for early-onset sepsis in preterm neonates and elucidate the pattern of nCD64 expression during bacterial infection. Secondly nCD64 expression was compared with conventional markers of neonatal sepsis. We also sought to assess the diagnostic utilities of its combination with other markers for early diagnosis of sepsis in preterm neonates.

## Materials and Methods

### Ethics statement

The study protocol was approved by the ethics committee of Shenzhen Shajing Hospital affiliated of Guangzhou Medical University prior to commencement and written informed consent was obtained from parents or legal representatives of children. For the use of surplus blood samples in control preterm neonates verbal consent from parents or legal representatives of children was obtained. No written consent was deemed necessarily for the use of surplus blood samples by the ethics committee. All parents received written information on the use of surplus blood samples for research purposes. The medical ethics committee of the Shenzhen Shajing Hospital affiliated of Guangzhou Medical University approved the consent procedure for the use of surplus blood samples.

### Patients

The prospective study was conducted in our hospital newborn intensive care unit (NICU). Consecutive preterm neonates (gestational age ≤36 weeks) undergoing sepsis evaluation were enrolled between November 2010 and June 2011. The length of antibiotic therapy varied on the basis of the severity of sepsis and the discretion of the neonatologist.

The preterm neonates were categorized into healthy control group and suspected sepsis group, based on the clinical features. Signs and symptoms suggestive of clinical sepsis have been described in detail in the previous studies [8], [11], [12]. As part of the evaluation, two or more of the following previously validated hematologic criteria were used as indicators for sepsis: (1) absolute neutrophil count of >12×10^3^ cells/mm^3^, (2) white blood cell count of >20×10^3^ cells/mm^3^, (3) immature/total neutrophil ratio of >20%, (4) platelet count of <150×10^3 ^cells/mm^3^, (5) CRP of >3.0 mg/L. The definitions of suspected sepsis were conducted blinded to biomarker data. For all the preterm neonates, the following data were collected: gestational age, gender, birth weight, length of stay and apgar scores.

Blood cultures were processed by Bactec microbial detection system (Becton-Dickinson, Sparks, MD). Chest radiograph was routinely performed during the initial screening procedure. Hematologic laboratory investigations including differential white blood cell count, platelet count, absolute neutrophil count were also performed. In addition to the routine serial CRP measurements, blood samples for neutrophil cell-surface antigens CD11b (nCD11b) and CD64 were obtained for evaluation. Preterm neonates (postnatal age <72 h) who had signs and symptoms suggestive of early-onset clinical infection or pneumonia requiring full sepsis evaluation, had 2 or more positive hematological criteria, and antibiotic treatment were defined as early-onset sepsis neonates. For each preterm neonates, the first sample was take at the onset of sepsis evaluation (within 12 hours after birth), and two further samples were obtained at the 24 hour and 72 hour after the onset. The two further samples were taken for clinically-indicated blood tests ordered by the treating physicians, and not done solely for the purposes of the study. The treating physicians were blind to the biomarker results. The clinical outcomes including the duration of treatment were not altered by the results of this study.

### Neutrophil CD64

Neutrophil CD64 expression was evaluated by flow cytometry (Becton Dickinson, Mountain View, CA, USA) using a phycoerythrin (PE) fluorescence quantification kit (QuantiBRITE PE, Becton Dickinson). Briefly, phosphate-buffered saline-diluted whole blood (50 µL) was incubated for 15 min at room temperature with a combination of anti-CD14-FITC and anti-CD64-PE. After lysis of red blood cells, samples were washed and fixed with BD Lyse/wash Assistant. Neutrophils were identified by electronic gating based forward and side scatter. The intensity of PE fluorescence on cells that were positive for anti-CD64-PE or anti-CD14-FITC was determined for a minimum of 10 000 cells. Inter-assay standardization and CD64 quantitation were performed by using QuantiBRITE PE calibration beads with known numbers of PE molecules. Data analysis was performed by using light scatter gating to define the neutrophil population, and the nCD64 value was quantified as mean equivalent soluble fluorescence units by using BD Diva software. Corrections for nonspecific antibody binding were performed by subtracting values for the isotype control. Expression of nCD11b was also measured by flow cytometry in the same way.

### Statistical Analysis

The results were analyzed using SPSS 17.0 statistical software (SPSS Inc, IL). The continuous variables for clinical parameters were expressed as means ± SEM. Comparisons between the sepsis and control groups were made using two-tailed t tests, and the *P* values were calculated. Receiver operating characteristic (ROC) curves were constructed and analyzed for the area under the curve (AUC). The optimal cut-off value was the closest point to the upper left-hand corner of the ROC curve, and enabled us to select the best marker or combination of markers at the most appropriate sampling time for diagnosing sepsis in preterm neonates. For each parameter, diagnostic usefulness was determined by calculating sensitivity, specificity, positive predictive value, negative predictive value. The AUC of different biomarkers was compared in Medcalc, version 11.5.1.0. Net reclassification improvement (NRI) was calculated to evaluate new biomarkers on their ability to increase the AUC [17]. A *P* value of <0.05 was considered significant.

## Results

A total of 158 preterm neonates were enrolled and studied, 88 and 70 of whom belonged to the suspected sepsis group and control group, respectively. The demographic data of the two groups is summarized in Table 1. Neonates with suspected sepsis (*n* = 88) were of lesser gestational age than those with no sepsis (*P*<0.001). Birth weight was also significant lower in suspected sepsis neonates compared with the controls (*P*<0.001). In addition, the apgar scores at 1 and 5 minutes differed significantly between the two groups. However, there were no differences in sex ratio and length of stay between the two groups (Table 1).

**Table 1:** Clinical characteristics of the study groups.

Variables	Suspected (*n* = 88)	Control (*n* = 70)	*P*
Gestational age, mean ± SEM (week)	31.60±0.44	33.78±0.31	0.000
Birth weight, mean ± SEM (g)	1598.63±88.43	2101.91±62.53	0.000
Male (female)	58 (30)	46 (24)	0.980
1-min Apgar score, median (range)	7 (5–10)	7 (7–10)	0.003
5-min Apgar score, median (range)	8 (6–10)	10 (9–10)	0.045
Length of stay, mean ± SEM (day)	20.54±2.26	15.67±1.87	0.102

**Figure 1:**
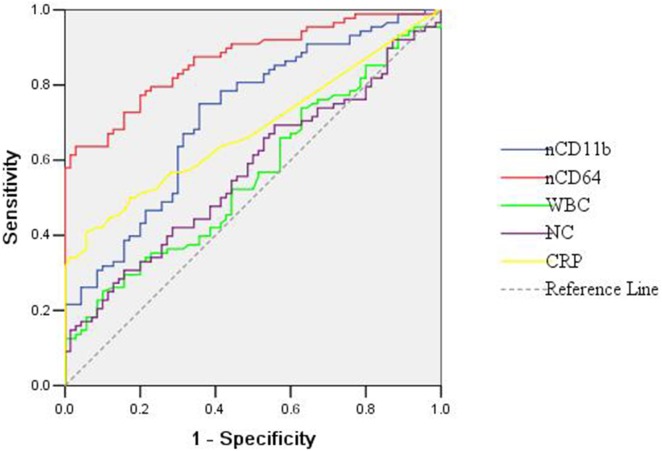
ROC curves of hematologic parameters in suspected preterm neonates. WBC: white blood cell count; NC: neutrophil count.

The levels of hematologic markers and neutrophil cell-surface antigens at the onset of sepsis evaluation are summarized in Table 2. The white blood cell count, neutrophil count and CRP were significant higher in suspected sepsis neonates compared with the corresponding values of controls (*P* = 0.018, 0.007 and 0.003, respectively). But the platelet count of the suspected sepsis neonates did not differ significantly from that of controls (*P*>0.01). Both nCD11b and nCD64 expression on neutrophils were evaluated by flow cytometry in 158 neonates. The nCD11b and nCD64 were significant higher in suspected sepsis neonates in comparison with controls (*P*<0.01). For all the useful parameters, ROC curves were constructed (Figure 1). The highest AUC was observed using nCD64 followed by nCD11b. The nCD64 had an AUC of 0.869, which was significant higher than that of white blood cell count, neutrophil count, CRP, and nCD11b (*P*<0.01) (Table 3); Use of a cutoff nCD64 value of 1010 PE-molecules bound/cell yielded a sensitivity of 81.82% and a specificity of 70.00%, with a negative predictive value of 75.4%. The sensitivity, specificity, positive predictive value, negative predictive value, and AUC of the useful parameters alone and in combination with the nCD64 (cutoff: 1010 PE-molecules bound/cell) are summarized in Table 3. Combination of the nCD64 value and neutrophil count performed a high sensitivity (85.23%) and negativity predictive value (78.7%). For the AUC, no significant differences were found between the nCD64 alone and the combination of two biomarkers (*P*>0.01) (Table 3). Reclassifications for subjects with and without suspected sepsis were conducted, and NRI was calculated to determine if neutrophil counts significantly improve discrimination when added to nCD64. The NRI was estimated at 0.042, and no significant difference was found between the AUC of nCD64 alone and the AUC of nCD64 plus neutrophil count (*P* = 0.543). Because the criteria for diagnosing sepsis in this study was ≥2 positive hematologic indices, the sensitivity and predictive values were not calculated for combinations of multiple hematologic parameters.

**Table 2:** Levels of biological markers at onset of sepsis evaluation.

Variables	Suspected (*n* = 88)	Control (*n* = 70)	*P*
White blood cell count, cell/mm^3^	10.96±0.61	9.25±0.35	0. 018
Platelet count, 1000 cells/mm^3^	227.58±10.75	235.70±11.03	0.600
Neutrophil count, 1000 cells/mm^3^	6.22±0.54	4.54±0.28	0.007
CRP, mg/L	3.68±0. 90	0.94±0.08	0.003
nCD11b, PE molecules bound/cell*	2869.67±205.19	1610.80±132.35	0.000
nCD64, PE molecules bound/cell*	2394.45±184.56	855.58±44.96	0.000

Results are mean ± SEM;

*PE: phycoerythrin.

**Table 3:** Sensitivity, specificity, and positive and negative predictive values of markers using optimal cutoff values.

Hematologic index^1^	Sensitivity (%, 95% CI)	Specificity (%, 95% CI)	PPV^2^ (%, 95% CI)	NPV^3^ (%, 95% CI)	AUC, mean ± SEM (95% CI)
WBC count^4^	73.86 (63.4–82.7)	37.14 (25.9–49.5)	59.60 (49.3–68.4)	53.10 (37.2–66.7)	0.559±0.046 (0.469–0.648)
Neutrophil count	65.91 (55–75.7)	47.14 (35.1–59.4)	61.1 (50.5–70.9)	52.4 (39.4–65.1)	0.571±0.045 (0.482–0.660)
CRP	40.91 (30.5–51.9)	94.29 (86.0–98.4)	90.0 (76.3–97.2)	55.9 (46.5–65.1)	0.680±0.042 (0.598–0.763)
nCD11b	75.00 (64.6–83.6)	64.29 (51.9–75.4)	72.5 (62.1–81.4)	67.2 (54.6–78.2)	0.717±0.041 (0.638–0.979)
nCD64	81.82 (72.2–89.2)	70.00 (57.9–80.4)	77.4 (67.6–85.4)	75.4 (63.0–85.3)	0.869±0.028 (0.814–0.923)
Neutrophil count and nCD64	85.23 (76.1–91.9)	68.57 (56.4–79.1)	77.3 (67.7–85.2)	78.7 (66.2–88.2)	0.871±0.027 (0.817–0.925)
CRP and nCD64	77.27 (67.1–85.5)	90.00 (80.5–95.9)	90.7 (81.6–96.2)	75.9 (65.3–84.6)	0.895±0.024 (0.847–0.942)
nCD11b and nCD64	73.86 (63.4–82.7)	94.29 (86.0–98.4)	94.2 (85.8–98.4)	74.2 (63.8–82.9)	0.896±0.025 (0.847–0.945)
WBC count and nCD64	82.95 (73.4–90.1)	72.86 (60.9–82.8)	79.3 (69.6–87.1)	77.3 (65.2–86.8)	0.872±0.027 (0.819–0.926)

1 CI, Confidence Interval;

2 PPV, positive predictive value;

3 NPV, negative predictive value;

4 WBC, white blood cell.

Of the 88 suspected sepsis neonates, pathogenic organisms could be isolated from 20 (23%) cases: 8 coagulase-netative *staphylococcus*, 4 *Klebsiella pneumoniae*, 4 *Stenotrophomonas maltophilia*, 3 *Escherichia coli*, 1 *Staphylococcus aureus*. The white blood cell count (*P* = 0.009) and neutrophil count (*P* = 0.003) were significantly higher in culture-positive suspected sepsis neonates than that in culture-negative suspected sepsis neonates (Table 4). However, the nCD64 (*P* = 0.849), nCD11b (*P* = 0.725) and CRP (*P* = 0.152) between the culture-positive suspected sepsis group and the culture-negative suspected sepsis group did not differ significantly. All the five parameters, white blood cell count, neutrophil count, CRP, nCD64 and nCD11b were significantly different between culture-positive suspected sepsis cases and controls (*P*<0.05) and between culture-negative suspected sepsis neonates and controls (*P*<0.05) except that the white blood count and neutrophil count were not statistically different between the culture-negative suspected sepsis neonates and controls (*P* = 0.217, 0.158; Table 4).

**Table 4:** Levels of biochemical markers and neutrophil cell-surface antigens at onset of sepsis in various groups.

Variables	Culture-positive suspected group (*n* = 20)	Culture-negative suspected group (*n* = 68)	Control (*n* = 70)	*P*
White blood cell count, cell/mm^3^	13.41±1.86	10.24±0.56	9.25±0.35	0. 003
Neutrophil count, 1000 cells/mm^3^	8.60±1.76	5.52±0.46	4.54±0.28	0.001
CRP, mg/L	5.47±2.79	3.16±0.83	0.94±0.08	0.010
nCD11b, PE molecules bound/cell*	2981.95±450.40	2836.65±231.86	1610.80±132.35	0.000
nCD64, PE molecules bound/cell*	2344.95±428.16	2409.01±204.78	855.58±44.96	0.000

*PE: phycoerythrin.

**Figure 2:**
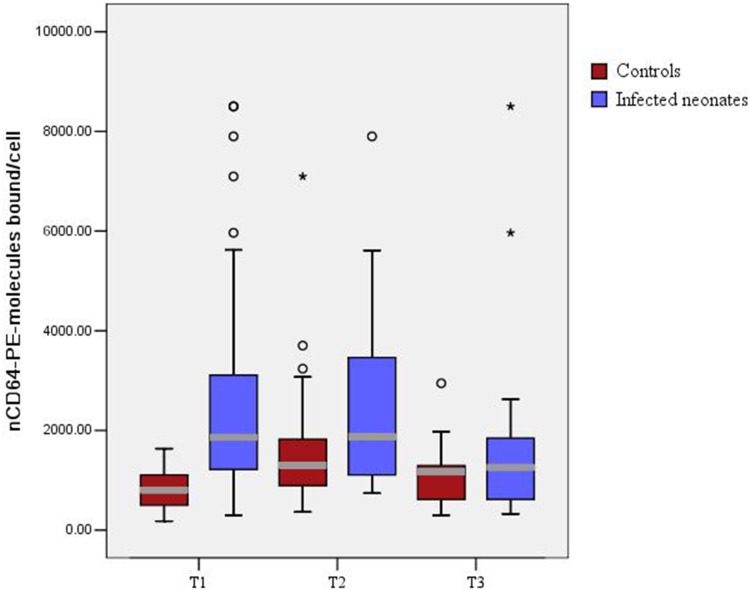
Boxplot distribution of the nCD64 in suspected group and control group. The *box* shows the 25^th^–75^th^ percentiles, whereas the *whiskers* indicate the 10^th^ and 90^th^ percentiles. T1, time at onset of sepsis (<12 hours after birth); T2, 12 hour after the onset of sepsis; T3, 72 hour after the onset of sepsis.

The pattern of expression of nCD64 during bacterial infection and its relation to antibiotic treatment were also investigated (Figure 2). In both suspected sepsis group and control group, nCD64 manifested its peak concentrations at approximately 24 hours after the onset (T2). For the suspected sepsis group, antibiotic therapy was given at the onset of sepsis (T1, approximately 12 hours after birth). Suspected sepsis neonates receiving adequate antibiotic therapy had quick reduction in their nCD64 value at about 72 hours after the onset (T3). For the control group, nCD64 was increased from T1 to T2, and then decreased from T2 to T3. At T1 and T2, the nCD64 in suspected sepsis group and control group was significantly different to each other. However, there was no difference in nCD64 expression between the suspected sepsis group and control group at T3 (Figure 2).

## Discussion

Sepsis is a major source of morbidity and mortality in the neonatal population. Early-onset sepsis of preterm neonates, diagnosed ≤72 hours after birth, is most often related to antenatal and perinatal factors [18]. The signs and symptoms of early-onset sepsis are subtle and nonspecific, particularly in preterm neonates. Isolation of pathogenic organism by culture is the gold standard for detection of neonatal sepsis. However, the blood culture results are not available rapidly, the best reported positive culture ratio reach only up to 50% [19]. For the fear of missing a true case of neonatal sepsis, antibiotics are administered to all suspected sepsis neonates [20]. This empiric therapy may result in antimicrobial overexposure, which promotes antimicrobial resistance and enhances neonatal healthcare cost.

Thus, the need for an early marker of neonatal sepsis with high sensitivity and specificity is readily apparent. Studies using peripheral/core temperature differences to identify neonates with sepsis have shown promise but have not been validated with larger numbers of infants [21]. Many studies have used hematologic parameters to increase the diagnostic efficiency for sepsis [22], [23]. However, variations in their reported cutoff values, methodologies, wide range of sensitivity and specificity preclude their diagnostic usefulness in clinical laboratories [24]. Cytokine levels in blood have also been investigated as a marker to increase the diagnostic efficiency for neonatal sepsis. Among the cytokines, most studies have confirmed the utility of interleukin-6 as an early marker of neonatal sepsis [25]–[27]. However, interleukin-6 is a very early marker, but levels can become normal even if infection continues [10]. This leads to an increasing proportion of false-negative findings when sampling is performed later in the course. Acute-phase reactants, such as CRP and procalcitonin have also been investigated as early indicator of neonatal sepsis [28]. While these markers have similar diagnostic efficiency, and no single marker has been found to be superior to the others.

The nCD64 is known as Fc-gamma receptor1 and expressed at a very low level on the surface of resting neutrophils [20]. There is a markedly increase in CD64 expression on the surface of neutrophils in response to bacterial infection in neonates, similar to that seen in older children and adults [14]. The levels remain high for 24 hours and have been shown to be independent of gestational age and antibiotic administration [29]. Thus, we evaluated the utility of nCD64 as a diagnostic marker for identifying early-onset sepsis in preterm neonates.

In this study, we found the expression of nCD64 was significantly upregulated in preterm neonates with suspected sepsis (*P*<0.001, Table 2). Significant lower birth weight and gestational age were also found in the suspected sepsis neonates (*P*<0.001, Table 1). Our results are similar with the previous studies. Soni et al. have reported increased expression of nCD64 in neonates with sepsis [20]. They found the monocyte/neutrophil CD64 ratio was a highly sensitive marker of culture-positive neonatal sepsis. Using ROC curves, Ng et al. have also reported increased expression of CD64 in both early-onset and late-onset neonatal sepsis [30], [31]. They found the nCD64 was a very sensitive marker for diagnosing nosocomial infection in very low birth weight infants. For the very low birth weight neonates, increase in expression of nCD64 was noted at the time of sepsis evaluation, and the level remained markedly raised at 24 h after the onset. Similar results were also found in the preterm neonates with early-onset sepsis in our study (Figure 2). These results further confirmed that the level of nCD64 in neonates was independent of antibiotic therapy within 24 h after the onset.

The nCD11b has also been previously suggested to be a highly effective marker for diagnosing early-onset infection (infection occurs within 24 hours of age) in neonates [32]. Our results support this notion. We found significant higher level of nCD11b expression in suspected sepsis neonates. In contrast, a previous study investigating more than 10 leukocyte surface markers was unable to confirm the diagnostic value of nCD11b for predicting infection in preterm infants [33]. The different efficiency of nCD11b in diagnosing neonatal sepsis might result from the different mechanism of early-onset infection and late-onset infection. In the early-onset infection, the pathogens are usually acquired within a short and well-defined period during peripartum [31]. However, one can never be certain in late-onset nosocomial sepsis at which phase of the infection blood was collected for determination of CD11b.

Many studies have evaluated the diagnostic utility of nCD64 as a marker in preterm neonatal sepsis [34]–[36]. Streimish et al. [36] evaluated the sensitivity and specificity of nCD64 as a diagnostic marker for clinical sepsis; In that study, 684 neonates were evaluated for sepsis, and the nCD64 has a sensitivity of 78% and a negative predictive value of 81%; While in combination with the absolute neutrophil count or the absolute band count, nCD64 had the highest sensitivity (91%) and specificity (93%), respectively. In our study, we found the nCD64 had the highest AUC and its sensitivity and negative predictive value was 81.82% and 75.4%, respectively. The conventional markers (white blood cell count, neutrophil count and CRP) and nCD11b were found to be less useful as markers diagnosing early-onset sepsis in preterm neonates. The combination of nCD64 and neutrophil count demonstrated 85.23% sensitivity and 78.7% negative predictive value, but the NRI showed no significant difference was found between the AUC of nCD64 alone and the AUC of nCD64 plus neutrophil count. Our results were similar with the previous studies, and these findings strongly suggest that the nCD64 could be efficacious in guiding decisions to withhold antibiotic therapy.

Blood culture was the “gold standard” for detection of systemic infection. Pathogenic organisms were isolated form 20 cases, and the isolated bacteria for early-onset sepsis are quite different to what is usually seen in Western countries. Previous studies have reported that the nCD64 was also a highly sensitive marker of culture-positive neonatal sepsis [8], [20], [37]–[39]. However, in our study, the nCD64 in culture-positive suspected sepsis group and culture-negative suspected sepsis group did not differ significantly. The different findings observed among different studies might be explained in several ways, such as the differences of the populations being evaluated or the technical differences in the methods of measurement among different trials.

The variations of nCD64 in response to bacterial infection and antibiotic therapy were also investigated (Figure 2). The changes of nCD64 level observed in the control group confirmed those previous reports that the level of nCD64 remained markedly high at 24 hour (T2) after the onset (T1) [31]. The changes of nCD64 level observed in the suspected sepsis group suggested that the level of nCD64 at 24 hour (T2) after the onset (T1) was independent of antibiotic treatment (Figure 2). This result was consistent with the previous studies [29], [39].

Neutrophil CD64 is a highly sensitive marker for the diagnosis of suspected early-onset sepsis in preterm neonates. The level of nCD64 is independent of antibiotic therapy within 24 hours after onset. Our findings suggest that nCD64 may be incorporated as a valuable marker to diagnose infection. In the future, nCD64 should be further evaluated and considered as a potential neonatal sepsis biomarker in routine clinical settings.
